# *Helicobacter pylori* eradication rate following first line non-bismuth quadruple therapy regimen in Tunisian patients: the HPERAD study

**DOI:** 10.1186/s12879-025-12405-0

**Published:** 2025-12-25

**Authors:** Monia Fekih, Taieb Jomni, Myriam Ayari, Hajer Battikh, Meriem Zribi, Asma Labidi, Meriem Serghini, Nadia Ben Zid, Marwa Hafi, Jalel Boubaker, Alaa Nciri, Mehdi Ben Abdelwahed, Mejda Zakhama, Mohamed Hichem Loghmari, Raoua Baklouti, Arwa Guediche, Wided Bouhlel, Imen Jemni, Asma Sabbek, Nabil Ben Chaabene, Leila Safer, Ons Haddad, Yosr Kadri, Hajer Rhim, Maha Mastouri, Emna Belhadj Mabrouk, Asma Mensi, Shema Ayadi, Yosra Zaimi, Yosra Said, Leila Mouelhi, Radhouane Debbeche, Mehdi Kchaou, Wafa Haddad, Lassaad Chtourou, Hamadi Chabchoub, Mohamed Turki, Mourad Njeh, Amine Mzid, Nesrine Medjahed, Basma Mnif, Adnene Hammami, Hatem Ben Abdallah, Ghanem Mohamed, Nabil Abdelli, Imen Abdelaali, Mohamed Hedi Douggui, Mouna Medhioub, Amal Khsiba, Moufida Mahmoudi, Asma Ben Mohamed, Manel Yakoubi, Lamine Hamzaoui, Mohamed Moussadek Azzouz, Norsaf Bibani, Dorra Trad, Meriam Sabbah, Houssaina Jlassi, Hela Elloumi, Dalila Gargouri, Bochra Bouchabou, Abdelwaheb Nakhli, Nesrine Hemdani, Rym Ennaifer, Leila Bel Hadj Ammar, Soumaya Nsibi, Raja Tlili, Lamia Kallel, Sonia Ben Hamida, Hanen Elloumi, Imed Cheikh, Mariem Nouira, Samir Ennigrou

**Affiliations:** 1 Department of Gastroenterology, A La Rabta Hospital, Tunis, Tunisia; 2 Department of Gastroenterology, Internal Security Forces Hospital La Marsa, Tunis, 2070 Tunisia; 3 Department of Microbiology, La Rabta Hospital, Tunis, Tunisia; 4 Department of Gastroenterology, Fattouma Bourguiba Hospital, Monastir, Tunisia; 5 Microbiology Laboratory, Fattouma Bourguiba University Hospital, Monastir, Tunisia; 6 Department of Gastroenterology, Charles Nicolle Hospital, Tunis, Tunisia; 7 Department of Gastroenterology, Avicenne Clinic, Tunis, Tunisia; 8 Department of Gastroenterology, Hédi Chaker Hospital, Sfax, Tunisia; 9 Department of Gastroenterology, Ibn Nafis Clinic, Sfax, Tunisia; 10 Department of Microbiology, Hédi Chaker Hospital, Sfax, Tunisia; 11 Department of Gastroenterology, Principal Military Hospital of Instruction of Tunis, Tunis, Tunisia; 12 Department of Gastroenterology, Mohamed Taher El Maamouri Hospital, Nabeul, Tunisia; 13 Department of Gastroenterology, Habib Thameur Hospital, Tunis, Tunisia; 14 Department of Gastroenterology, Mongi Slim Hospital, Tunis, Tunisia; 15 Department of Gastroenterology, Mahmoud El Matri Hospital, Ariana, Tunisia; 16 Department of Gastroenterology, Habib Bougatfa Hospital, Bizerte, Tunisia; 17 Preventive Medicine Department, Faculty of Medicine of Tunis, Tunis, Tunisia

**Keywords:** *Helicobacter pylori*, Concomitant quadruple therapy, Non-bismuth therapy, Eradication, First-line treatment, Multicenter, Prospective study

## Abstract

**Introduction:**

Helicobacter pylori (H. pylori) eradication is a continuously challenging issue as antibiotic resistance is evolving and adversely affecting outcomes of previously effective treatments. Continual assessment and modification of therapeutic approaches is necessary.

**Aim:**

The aim of our study was to evaluate H. pylori eradication rate in Tunisian patients treated by non-bismuth quadruple therapy and to investigate treatment failure factors.

**Methods:**

We conducted a prospective multicentric study including patients with H. pylori infection. For all patients, concomitant therapy (amoxicillin 1 g, clarithromycin 500 mg and metronidazole 500 mg plus esomeprazole 40 mg) twice daily for 14 days was prescribed. Mutations conferring resistance of H. pylori to clarithromycin were detected using polymerase chain reaction on gastric biopsies. Eradication was assessed using the urea breath test.

**Results:**

414 patients were included. The mean age was 44.7 ± 14.3 with a sex ratio of M/F = 0.53. Mutations conferring resistance of H. pylori to clarithromycin were observed in 76 patients (18.35%). During follow-up, adverse effects to treatment occurred in 34.5% of patients. Eradication rate was 88.2% (95% CI: [85.1–91.3]) in per protocol analysis. In multivariate analysis, female gender (OR:2.924, 95% CI (1.340–6.380), *p* = 0.009) and the presence of clarithromycin resistance gene (OR:4.671, 95% CI (2.444–8.925), *p* < 0.001) were predictors of treatment failure.

**Conclusion:**

Concomitant therapy showed an eradication rate below 90% in Tunisian patients, mainly due to clarithromycin resistance and female gender. However, non-bismuth quadruple therapy remains a viable alternative option, achieving eradication in more than 8 out of 10 patients.

**Clinical trial:**

Not applicable

## Introduction

*Helicobacter pylori* (H. pylori) is a microaerophilic, Gram-negative bacillus that colonizes the human gastric mucosa. Transmission is mainly inter-human, via the oro-oral or fecal-oral routes. Contamination occurs in childhood, mainly during the first five years of life, rarely in adulthood. H. pylori infection is globally widespread. Favored by poor hygiene and low socioeconomic conditions, it remains more common in developing countries ( > 70% of adults) than in developed countries ( < 40–50%) [1]. Recently a systematic review and meta-analysis showed a decrease in the global prevalence of H. pylori between 1980 and 90 – 2011–2022 from 58.2% to 43.1% respectively, with the most pronounced decrease observed in Africa, (85.1% versus 53.3%, respectively) [2]. Despite this reduction, prevalence rates remain high. In Tunisia, first studies demonstrated that the prevalence in asymptomatic adult population was about 82.7% [3], up to 89% in symptomatic patients [4] and 51.4% among school children in the first year of primary school [5]. More recently, an epidemiological study conducted in 2020 (unpublished data), including 1800 blood donors in multiple centers (Tunis, Sfax, Sousse, Gafsa and Jendouba), found positive H. pylori IgG antibodies in 71.7% of participants [6]. In addition, a Tunisian multicenter study conducted in 2022, showed that H. pylori infection was identified in 60.9% of symptomatic patients undergoing upper gastrointestinal endoscopy, using gene amplification test by *polymerase chain reaction* (PCR), and in 57.2% by histopathological analysis [6], which is in line with the global trend. These findings highlight the continual need for ongoing efforts to achieve eradication and reduce the burden of gastric cancer, as H. pylori is classified by the World Health Organization (WHO) as a Group 1 human carcinogen [7]. The importance of eradication has been further demonstrated by its significant impact in reducing the incidence and mortality of gastric cancer [8]. However, the management of H. pylori infection has become increasingly challenging due to growing antibiotic resistance. Multiple antibiotic regimens have been evaluated, with a frequent decline in their efficacy over time. Consequently, the conventional triple therapy combining PPI, clarithromycin, and either amoxicillin or metronidazole is no longer recommended due to unacceptably low eradication rates [9]. This decline can be attributed to several factors, including poor patient compliance, high antibiotic resistance, specific bacterial characteristics, and host genetic polymorphisms.

Ongoing updates of treatment guidelines reflect the evolving landscape of H. pylori management. The choice of different empirical treatment regimens is essentially guided by local antibiotic resistance patterns. According to the latest Maastricht recommendations, in regions with strong resistance to antibiotics, such as Tunisia with a reported primary clarithromycin resistance rate of 25.3% [10], concomitant therapy, with or without bismuth, should be prescribed as the first-line empirical treatment for H. pylori eradication [11]. Indeed, although many empirical regimens show high failure rates in areas with substantial clarithromycin resistance, non-bismuth concomitant therapy has emerged as a promising alternative, owing to its synergistic antibiotic combination [12]. Based on that, the non-bismuth regimen was commonly prescribed in Tunisia as widely available. However, recent robust region-specific data regarding its efficacy in Tunisian populations have been lacking.

The aim of our study was to evaluate the effectiveness of non-bismuth quadruple therapy (esomeprazole, amoxicillin, and clarithromycin) in the context of a high rate of clarithromycin resistance. Specifically, we sought to determine the eradication rate following concomitant therapy in Tunisian patients and to identify factors associated with treatment failure including the presence of primary clarithromycin resistance.

## Material and methods

We conducted a prospective interventional longitudinal multicentric study from October 2021 to August 2022 involving twelve endoscopy centers in collaboration with three laboratories of bacteriology, among symptomatic patients requiring upper endoscopy and to whom the search for H. pylori was indicated, according to the European recommendations of Maastricht IV [13].

### Patients

We included patients aged ≥18 years with confirmed H. pylori infection, documented by gastric biopsy through bacteriological tests (rapid *urease* test, direct examination, culture, PCR) or histology and with no prior eradication therapy.

Non-inclusion criteria were refusal to provide informed consent, antibiotic use within the month prior to inclusion, history of gastric surgery, celiac disease, renal or hepatic insufficiency, gastric or intestinal neoplasia, gastric ulcers or complicated duodenal ulcers, pregnancy or breastfeeding, and allergy to amoxicillin or clarithromycin.

Patients who received incomplete treatment equal to or less than 80% of the prescribed dose or duration, were lost to follow-up, or developed an allergy to amoxicillin or clarithromycin during the study were excluded.

### Study protocol

* Pre-selection visit: Patients were interviewed at the endoscopy units of the investigating centers participating in the study to verify eligibility criteria and then had the scheduled upper endoscopy. This examination was performed by the investigating physicians, with 4 biopsies done for bacteriological work-up and 5 biopsies for pathological examinations.

* Inclusion visit: Once H. pylori infection was confirmed (3 to 21 days after pre-selection visit), eligible patients were included and received eradication treatment with non-bismuth quadruple regimen combining a double dose proton-pump inhibitors (PPI): esomeprazole (40 mg twice daily), clarithromycin (500 mg twice daily), metronidazole (500 mg twice daily) and amoxicillin (1 g twice daily) for 14 days.

* Control visit: On days 5 and 7, patients received a phone call to assess adherence and tolerance. At week 8 after completion of the H. pylori eradication regimen, all patients attended a follow-up visit during which clinical symptoms, occurrence of adverse effects, and treatment compliance were evaluated. All patients were referred for a ^13^C-urea breath test (^13^C-UBT), performed at least two weeks after discontinuing PPI, to investigate H. pylori eradication.

* End-of-study visit at week 12: Patients received the breath test result. Two situations raised: successful eradication (negative breath test) or persistence H. pylori infection (positive breath test). In the latter situation, patients received second-line treatment, guided by the antibiogram result when available.

**Fig. 1:**
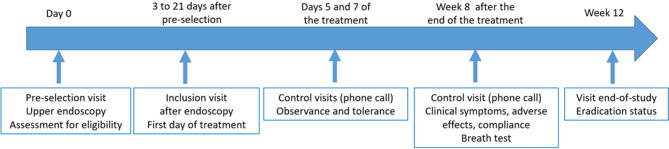
The design of the study

The design of the study is illustrated in Fig. 1.

### Bacterial analysis

Gastric biopsies were obtained via endoscopy and placed in a sterile, airtight container containing either physiological saline or a transport medium (Pylorigerm®, Biomérieux). The biopsies were then minced into small fragments using two scalpels in a Petri dish under sterile conditions. The fragments were processed as follows:

- Urease activity test: A portion of the minced material was placed in a tube containing 1 ml of urea-indole solution. The sample was incubated at 37 °C for 24 hours to test for urease activity.
- Gram staining: One fragment was used for direct examination by Gram staining.
- Culture: A fragment was cultured on the following media: Blood agar, Columbia blood agar with horse blood supplemented with Skirrow® mix (Thermo Scientific), Selective Pylori Agar® (Biomérieux). The cultured media were placed in a microaerophilic jar (GENboxmicroaer®) at 37 °C for a minimum of 5 days. In the case of a positive culture, an antibiogram was performed on Mueller-Hinton agar supplemented with 10% horse blood, according to CA-SFM-EUCAST 2022 guidelines. The following antibiotics were tested using E-test strips (Biomérieux®): clarithromycin, levofloxacin, tetracycline, rifampicin, amoxicillin, metronidazole.
- Detection of clarithromycin resistance: Another fragment was used to detect mutations conferring resistance of H. pylori to clarithromycin by real-time PCR. DNA extraction was performed using the QIAamp DNA Kit® (QIAGEN), and amplification was carried out with the Allplex® Kit (Seegene).

- Number of subjects required: the sample size was determined after a pilot phase of 100 patients, with the aim of achieving statistical power of 80% or more. During this phase 90 samples were analyzed for the presence of H. pylori resistance genes to clarithromycin using PCR. Among the 63 H. pylori-positive samples, 14 (22.2%) showed clarithromycin resistance. Using this estimated prevalence (*p* = 0.222), the minimum sample size required to achieve 80% statistical power with a 95% confidence level (z = 1.96) and a 4% margin of error (i = 0.04). The calculated sample size, using the following *n* = z^2^ x p (1 – p)/i^2^, was *n* = 415.

- Data collection: the variables collected were divided into demographic variables, social data, body mass index (BMI), comorbidities, symptoms, upper endoscopy and histology findings, bacteriological work-up, potential adverse events.

- Statistical analysis: The overall eradication rates and their 95% confidence intervals were obtained by intention to treat (ITT) and per protocol (PP). Quantitative variables were given as mean ± standard deviations and qualitative variables were presented as percentages. A univariate analysis was performed using the chi-squared test or the Mann–Whitney *U*-test. The significance level was set at *p* < 0.05 for all statistical tests. Variables significant at 20% in univariate analysis were introduced into binary logistic regression for a multivariate analysis. Calculations were performed using the SPSS 21 software.

**Fig. 2:**
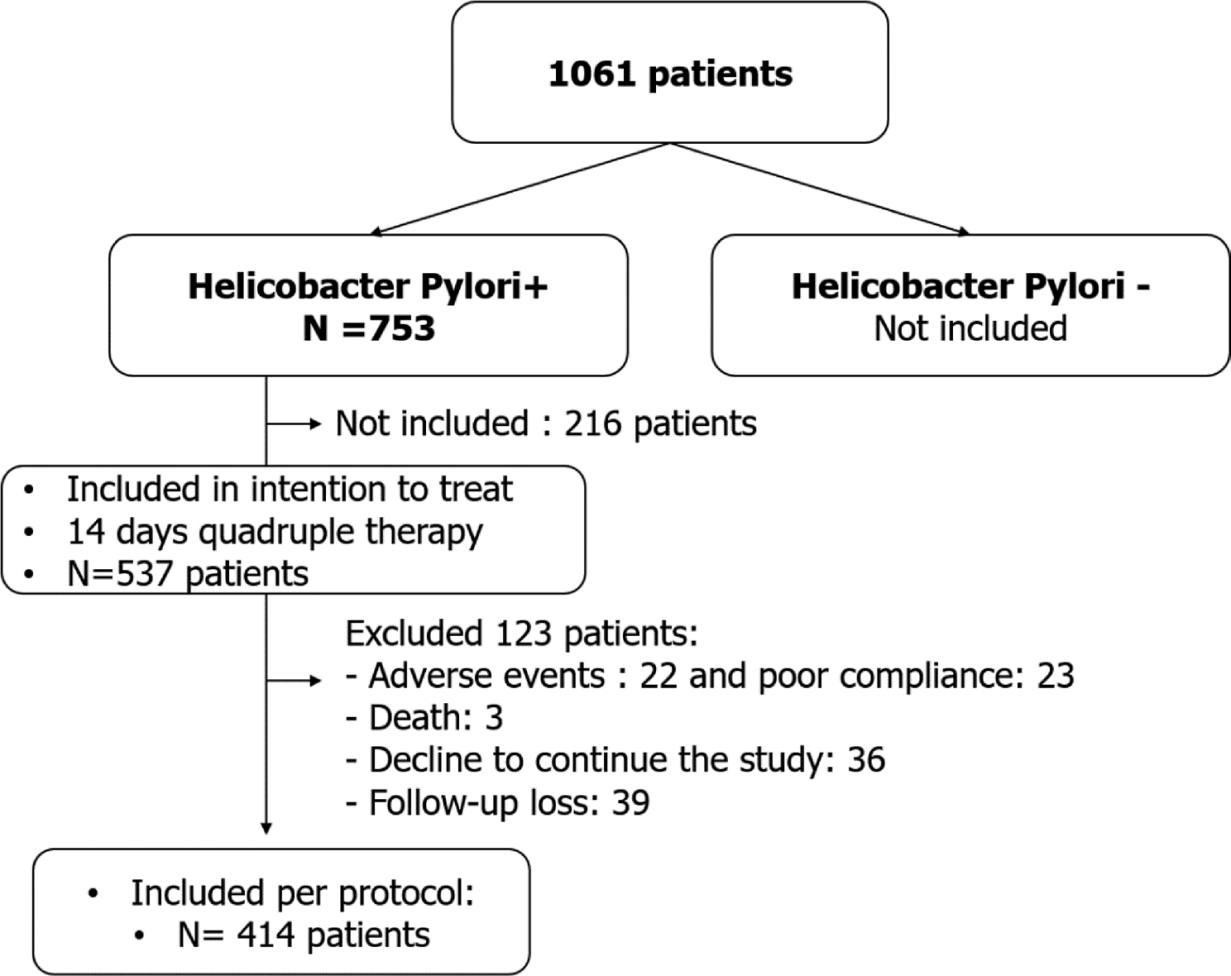
Flowchart of the study

Results: In total, 414 H. pylori infected patients were included (Fig. 2).

The mean age of the participants was 44.7 ± 14.3 with a minimum and maximum age of 18 years and 75 years respectively and a sex ratio of M/F = 0.53. The mean BMI was 25.4 ± 2.5 kg/m^2^. Among the patients, 21.5% (*n* = 89) were active smokers and 7% (*n* = 29) were consuming alcohol. Regarding comorbidities, diabetes and heart disease were found in 8.5% (*n* = 35) and 2.5% (*n* = 10) respectively. The main indications of upper endoscopy were epigastric pain (80.2%), gastro esophageal reflux (31.6%), vomiting (14.7%), and anemia (13%). General, endoscopic, histologic characteristics and H. pylori diagnostic method are described in Table 1.

**Table 1:** General, endoscopic, histologic and bacteriological characteristics of included patients

Variable	ITT patients	PP patients
	N (%)	N (%)
**Age**		
< 50 years	328 (61.1%)	242 (58.5%)
≥50 years	209 (38.9%)	171 (41.5%)
**Gender**		
Female	335 (62.4%)	269(65.0%)
Male	202 (37.6%)	145 (35.0%)
**Education status**		
Unschooled	22 (4.1%)	19 (4.6%)
Primary	91 (16.9%)	73 (17.6%)
High school	193 (35.9%)	159 (38.4%)
University	145 (27%)	105 (25.4%)
Unspecified	86 (16.1%)	58 (14%)
**Body mass index (kg/m**^**2**^)		
-Underweight ( < 18.5)	6 (1.1%)	5 (1.2%)
-Normal (18.5 to 24.9)	75 (14%)	58 (14%)
-Overweight: (25 to 29.9)	434 (80.8%)	334 (80.7%)
-Class I obesity: (30 to 34.9)	16 (3%)	12 (2.9%)
-Class II obesity: (35 to 39.9)	6 (1.1%)	5 (1.2%)
-Class III obesity: (≥40)	0	0
**Endoscopic findings**		
-Erythematous gastropathy	316 (58.8%)	242 (55.5%)
-Nodular gastropathy	148 (27.6%)	116 (28%)
-Duodenal ulcer	43 (8%)	37 (8.9%)
-Erosive gastropathy	35 (6.5%)	27 (6.5%)
-Esophagitis	41 (7.6%)	25 (6%)
-Atrophic gastropathy	13 (2.4%)	10 (2.4%)
**Histological findings:**		
-Gastritis	461 (85.8%)	364 (87.9%)
-Antral atrophy	85 (15.8%)	70 (16.9%)
-Fundic atrophy	58 (10,8%)	47 (11,4%)
-Fundic intestinal metaplasia	18 (3.4%)	14 (3.4%)
-Antral intestinal metaplasia	32 (6%)	28 (6.8%)
**H. Pylori density**:		
-Low	155 (32.8)	118 (31.5)
-Moderate	224 (47.4%)	178 (46.6%)
-High	94 (19.9%)	78 (20.8%)
**H. Pylori positivity diagnostic method**		
-Rapid *urease* test	296 (55.5%)	227 (54.8%)
-Direct examination	225 (41.9%)	167 (40.3%)
-Culture	11 (2%)	10 (2.4%)
* -Polymerase chain reaction* (PCR)	307 (57.2%)	260 (62.8%)
-Histology	473 (88.3%)	375 (90.6%)

Mutations conferring resistance of H. pylori to clarithromycin were observed in 76 patients (18.35%). During follow-up, adverse effects to treatment occurred in 34.5% of patients reported as trouble in taste/smell (16.2%), epigastric pain (13.5%), nausea/vomiting (6.8%), asthenia/anorexia (5.3%), diarrhea (4.8%), dizziness (3.6%), itching (0.7%), abdominal bloating (0.7%) and cephalalgia (0.7%).

Of the 414 patients treated and followed, eradication attested by negative ^13^C-UBT was successful in 365. Eradications rates were 88.2% (95% CI: 85.1–91.3) and 68% (95% CI: 63.5–72.5) by per-protocol and intention to treat analysis respectively.

The eradication rate was not significantly different according to gender, age, demographic data, geographic origin, smoking and alcohol drinking status. In the same way, comorbidities, endoscopic findings, histologic characteristics, H. pylori intensity and adverse effect were not correlated to treatment failure in our study. Associated factors with eradication failure in univariate analysis are summarized in Table 2.

**Table 2:** Associated factors with treatment failure in univariate analysis

	Success eradication	Failure eradication	p
	(n = 365)	(n = 49)	
**Age**			
< 50 years	219 (90.5%)	23(9.5%)	
> 50 years	156 (84.9)	26 (15.1%)	0.082
**Gender**			
Female	229 (85.1%)	40 (14.9)	0.009
Male	136 (92.8%)	9 (6.2%)	
**Drinking water**			
Yes	325 (87.6%)	46 (12.4%)	0.297
No	40 (90%)	3 (10%)	
**Active smoking**			
Yes	83 (93.3%)	6 (6.7%)	0.093
No	282 (86.8%)	43 (13.2)	
**Alcohol intake**			
Yes	27 (93.1)	2 (6.9%)	0.556
No	338 (87.8%)	47 (12.2%)	
**Diabetes mellitus**			0.588
Yes	30 (85.7%)	5 (14.3%)	
No	335 (88.4%)	44 (11.6%)	
**Obesity**			
Yes	15 (88.2%)	2 (11.8%)	0.999
No	350 (88.2%)	47 (11.8%)	
**Overweight**			
Yes	306 (87.2%)	45 (12.8%)	0.143
No	59 (93.7)	4 (6.3%)	
**Erythematous gastropathy**			
Yes	215 (88.8%)	27 (11.2%)	0.612
No	150 (87.2%)	22 (12.8%)	
**Nodular gastropathy**			
Yes	101 (87.1)	15 (12.9)	0.667
No	264 (88.6%)	34 (11.4%)	
**Duodenal ulcer**			
Yes	34 (91.9%)	3 (8.1%)	0.6
No	331 (87.8%)	46 (12.2%)	
**Gastritis**			0.15
Yes	324 (89%)	40 (11%)	
No	41 (82%)	9 (18%)	
**Gastric antral atrophy**			
Yes	39 (83%)	8 (17%)	0.486
No	326 (88.8%)	41 (11.2%)	
**Fundic Intestinal metaplasia**			
Yes	353 (88.3%)	47 (11.8%)	0.676
No	12 (85.7%)	2 (14.3%)	
**Antral Intestinal metaplasia**			
Yes	23 (82.1%)	5 (17.9%)	0.357
No	342 (88.6%)	44 (11.4%)	
**Hp density**			
High	66 (84.6%)	12 (15.4)	
Moderate	160 (89.9%)	18 (10.1%)	0.364
Low	107 (90.7%)	11 (9.3%)	
**Adverse effect**			
Yes	87 (89.7%)	10 (10.3%)	0.595
No	278 (87.7%)	29 (12.3%)	
**Clarithromycin resistance**			
Yes	54 (71.1%)	22 (28.9%)	** < 0.001**
No	311 (92%)	27 (8%)	

Logistic model analysis included age > 50 years, female gender, active smoking, overweight, gastritis and clarithromycin resistance. Multivariate analyses demonstrated that female gender and resistance to clarithromycin were significantly related to eradication failure (Table 3).

**Table 3:** Predictors of helicobacter eradication failure in multivariate analysis

Variable	OR	95 CI	P
Resistance to clarithromycin	4.671	2.444–8.925	*<*0.001
Female gender	2.924	1.340–6.380	0.009

## Discussion

Eradication treatment of H. pylori has been the subject of numerous developments through the years. Successful eradication is based on a challenging resolution of a difficult equation, considering geographical considerations, bacterial strains, emerging resistance and individual tolerance. The initial diagnostic strategy in our study population involved gastroscopy. In Tunisia, where H. pylori prevalence remains high, the relevance of a non-invasive ‘test-and-treat’ approach may be discussed. However, given the frequent presence of symptoms and the rising antibiotic resistance, endoscopy-based diagnosis continues to be widely practiced. Treatment should be adapted based on antibiotic susceptibility. Ideally, the most effective approach is to adapt treatment based on antibiotic susceptibility. However, H. pylori sampling for bacteriological purposes is not routinely performed due to time and cost limitations. H. pylori infection is most often discovered incidentally on pathological samples, highlighting the importance of assessing and adapting empiric treatment. Given the decline of triple therapy and the complexity of sequential treatment, non-bismuth concomitant therapy of amoxicillin, metronidazole, clarithromycin and PPI appeared to be an effective, safe, and well-tolerated alternative [14] and recommended as first line therapy by different guidelines [11, 13, 15, 16]. In Tunisia, a monocentric published study have found an eradication rate of 81.6% [17]. Ethnicity and geographic regions play significant roles in determining the effectiveness of antibacterial treatments. Thus, investigating H. pylori eradication rates in different regions is necessary. Eradication rate superior to 90% is considered the optimal cut-off for PP analysis and a major goal for all regimens. In our study, the PP eradication rate was about 88% which is considered by Graham et al. as borderline acceptable (85–89%) [18]. Various PP eradication rates of non-bismuth quadruple therapy have been reported in the literature ranging from 77% to 98.5% [17, 19–28].

A recent systematical review including 6,906 patients and assessing the efficacy of non-bismuth regimen, have found an overall eradication rate of 87% in agreement with our results [29]. Usually, H. pylori eradication rate is associated with patient drug compliance and antibiotic resistance. Our findings identified clarithromycin resistance and female gender as independent predictors of treatment failure. Some studies reported that female gender may impact the eradication of H. pylori and was found to be associated with eradication failure following eradication therapy [30–32] with a 1.73-fold higher risk of failure compared to males [32]. This result could be explained by potential differences in gastric physiology between males and females that would impact colonization and response to treatment [33]. Indeed, females may have lower gastric acid output, which can impair the activation of acid-dependent antibiotics influencing treatment success [34]. Other hypothesis is that female may be more susceptible to infection with specific H. pylori strains, particularly those with A2143G mutations in the 23S rRNA gene, which is associated to clarithromycin resistance [35].

Few studies have prospectively investigated antibiotic resistance susceptibility and eradication rates following non-bismuth quadruple therapy as in our study. Taiet al. [20] have found that clarithromycin resistance, metronidazole resistance as well as dual clarithromycin and metronidazole resistances were the clinical factors influencing H. pylori eradication in the group of non-bismuth quadruple therapy. In others studies, poor compliance, adverse effects and advanced age were associated to eradication failure [23, 24, 26]. In our study, we have excluded patients with poor compliance and only clarithromycin resistance and female gender were found to correlate with treatment failure reinforcing that antibiotic resistance is the most serious issue in the cure of H. pylori. In Tunisia, the first bacteriological studies on H. pylori antibiotics resistance date back to 2006–2007 [36]. According to these studies, the rates of resistance to clarithromycin and metronidazole were about 15.4% and 51.3% respectively. Double resistance to clarithromycin and metronidazole was 13.6% [36]. Recently, Chtourou et al. reported a primary resistance rate to clarithromycin of 25.3% in a cross-sectional study of 101 PCR-positive H. pylori patients. The most frequently detected mutations were A2143G (*n* = 86, 90%) followed by A2142G (*n* = 11, 36%) [10]. Clarithromycin resistance increasing rate may explain the observed non-optimal eradication rate in our study. Indeed, Huang et al. demonstrated that the efficacies of non-bismuth containing quadruple therapies in the treatment of first-line against H. pylori across 4-year time interval (2013–2017) decreased as clarithromycin resistance increased [37]. Although 14-day concomitant therapy is to be considered fist-line treatment even in regions where H. pylori is highly resistant to Clarithromycin (above 15%) [11, 15, 26], our results showed an H. pylori eradication rate less than 90%. In the light of this results and considering the suboptimal response of concomitant therapy in this study, recent Tunisian H. pylori consensus have recommended bismuth regimen as first line empiric treatment [6]. Bismuth resistance is rarely reported and the addition of bismuth to eradication therapy would improve treatment results in areas with increasing resistance to clarithromycin, overcoming the consequent decrease of the antimicrobial efficacy. In fact, a recent systematic review and meta-analysis showed that bismuth containing therapy was the most effective in increasing eradication rates of dual-resistant H. pylori strains, followed by clarithromycin- and metronidazole-resistant strains [38].

This study has some limitations that should be acknowledged. The investigation focused on investigating clarithromycin resistance, without evaluating other resistance patterns of key antibiotics involved in the concomitant regimen. This may limit the ability to fully interpret the impact of multi-drug resistance on H. pylori eradication outcomes. In addition, the absence of a control group represents a significant limitation. Without a direct comparison with alternative treatment such as bismuth-based therapy, our findings cannot conclusively confirm the superior efficacy of the bismuth treatment in the Tunisian population. Future randomized controlled trials are warranted to validate our findings and to identify the most effective first-line therapy in a region with high antibiotic resistance.

In conclusion, fourteen days concomitant therapy could be considered as a relatively acceptable first line option in areas with high clarithromycin resistance. Bismuth regimen is to be considered in first line treatment as a promising alternative overcoming the evolving anti-microbial resistance and should be evaluated in Tunisian patients in further studies. Our study highlights the need to adopt a dynamic approach and to continuously optimize current H. pylori eradication regimen in each country, considering the local antibiotic susceptibility and bacterial ecology in order to improve treatment outcomes.

## Data Availability

The authors confirm that the data supporting the findings of this study are available within the article.

## Abbreviations

H. pylori: Helicobacter pylori
PCR: Polymerase chain reaction
PPI: Proton-pump inhibitors
^13^C-UBT: ^13^C-urea breath test
BMI: Body mass index
ITT: Intention to treat
PP: Per protocol
